# Effect of 980 nm diode laser irradiation in comparison with conventional irrigation on smear layer removal from radicular dentin—an in vitro experimental study

**DOI:** 10.1038/s41405-026-00409-0

**Published:** 2026-03-02

**Authors:** Syeda Abeerah Tanveer, Robia Ghafoor, Adil Omerson

**Affiliations:** 1 Operative Dentistry and Endodontics, Department of Dentistry, Aga Khan University Hospital, Karachi, Pakistan; 2 Master in Laser Dentistry, Karachi, Pakistan

**Keywords:** Endodontics, Root canal treatment

## Abstract

**Introduction:**

The smear layer in radicular dentin reduces effective disinfection by occluding dentinal tubules and decreasing dentin permeability, contributing to persistent microbial infection and root canal treatment failures.

**Objective:**

To compare the effect of 980 nm Diode laser irradiation and conventional irrigation with Sodium hypochlorite and ethylenediaminetetraacetic acid (NaOCl +EDTA) on smear layer removal in radicular dentin through dye penetration test.

**Material and methods:**

Sixty-six extracted single-rooted permanent teeth were randomly allocated into two groups. Group I underwent 980 nm diode laser irradiation using a 200 µm fiber in helicoidal motion (2 W power, 200 Hz frequency, 1–4 ms pulse duration). Group II received conventional irrigation with 3% NaOCl followed by 17% EDTA. All specimens were immersed in 2% methylene blue dye for 48 h, after which cross-sections were obtained at 3, 5, and 8 mm from the anatomical apex. Dye penetration diameter (mm) and area (mm²) between the inner and outer circumferences were measured using ImageJ software under a stereomicroscope. Statistical analysis was performed using one-way ANOVA with Bonferroni post-hoc tests.

**Results:**

The diode laser irradiation demonstrated overall significantly greater dentinal tubule penetration (16.2 ± 1.91 mm) compared to the conventional irrigation (5.32 ± 0.70 mm; *p* = 0.001). The overall mean area of the laser group (12.61 ± 2.02 mm²) was greater as compared to the conventional group (1.67 ± 0.73 mm²; *p* = 0.001).

**Conclusion:**

Diode laser irradiation may serve as an effective adjunct for smear layer removal and improved root canal disinfection.

## Introduction

The long-term success of endodontic treatment is fundamentally dependent on the thorough eradication of microorganisms from the complex root canal system [1, 2]. This is primarily achieved through a combination of effective irrigation and meticulous instrumentation protocols designed to clean and shape the canals [3]. Despite these measures, the process of mechanical instrumentation inevitably produces a smear layer, which is typically 1–5 μm thick [4]. This layer is composed of dentin debris, pulp tissue remnants, and bacterial by-products, and it has the ability to occlude dentinal tubules to depths of up to 40 μm, forming what are known as smear plug [5]. The presence of this smear layer presents a significant obstacle to achieving complete canal disinfection by sealing off dentinal tubules, reducing dentin permeability, and preventing the penetration of intracanal medicaments, resins, and sealers into deeper dentin layers [6]. As a result, the smear layer compromises the hermetic seal of the canal, which is essential for preventing reinfection and ensuring long-term treatment stability [7]. This makes smear layer removal a critical step in endodontic therapy.

To address this, several contemporary strategies have been developed. Advanced dentin conditioning techniques have been proposed to chemically alter the smear layer and enhance tubule exposure [8, 9]. Among the various chemical agents utilized in routine root canal therapy, sodium hypochlorite (NaOCl) continues to be the irrigant of choice due to its broad-spectrum antimicrobial action and ability to dissolve organic tissue at the concentrations ranging from 0.5 to 5.25% [10]. Complementing this, ethylenediaminetetraacetic acid (EDTA) is widely employed as a chelating agent, capable of dissolving the inorganic components of dentin, thereby exposing dentinal tubules and enhancing dentin permeability [11]. A study by Crumpton et al. [12] and Ballal et al. [13] demonstrated that the conventional protocol involving NaOCl and EDTA facilitates removal of the smear layer by chelating calcium ions from dentin. Despite their efficacy, the performance of NaOCl and EDTA is highly dependent on key variables such as concentration, pH, and duration of exposure [8]. These drawbacks highlight the importance of optimizing irrigation protocols to balance effective smear layer removal with preservation of dentin structural integrity.

Contemporary treatment modalities incorporated photodynamic therapy (PDT) with lasers as an intracanal disinfection has been extensively investigated in recent years [14]. Among the different laser systems, diode lasers have gained particular attention due to their ability to achieve effective microbial reduction within the root canal system [15]. Their wavelength-specific absorption properties enable efficient disinfection with minimal thermal or structural damage to dentin and surrounding hard tissues [16]. Previous studies have shown an antibacterial effect of 810- to 980-nm diode lasers on standard bacterial strains associated with endodontic infection [17, 18]. Despite substantial advancement in modern endodontics, post-treatment failures remain remarkably prevalent, owing to the persistence of microbial remnants in the smear layer [19]. The impact of PDT-mediated diode lasers on smear layer removal and patency of root canal dentin is still unprecedented and requires further investigation to determine its effectiveness. Thus, this study aims to evaluate and compare the effect of 980 nm diode laser irradiation and conventional irrigation using 3% NaOCl+ 17% EDTA on smear layer removal in radicular dentin through a dentin permeability/ dye penetration test. By generating the null hypothesis that there will be no difference in the effectiveness of diode laser and conventional irrigation on the smear layer in root canal dentin.

## Materials and methods

**Fig. 1:**
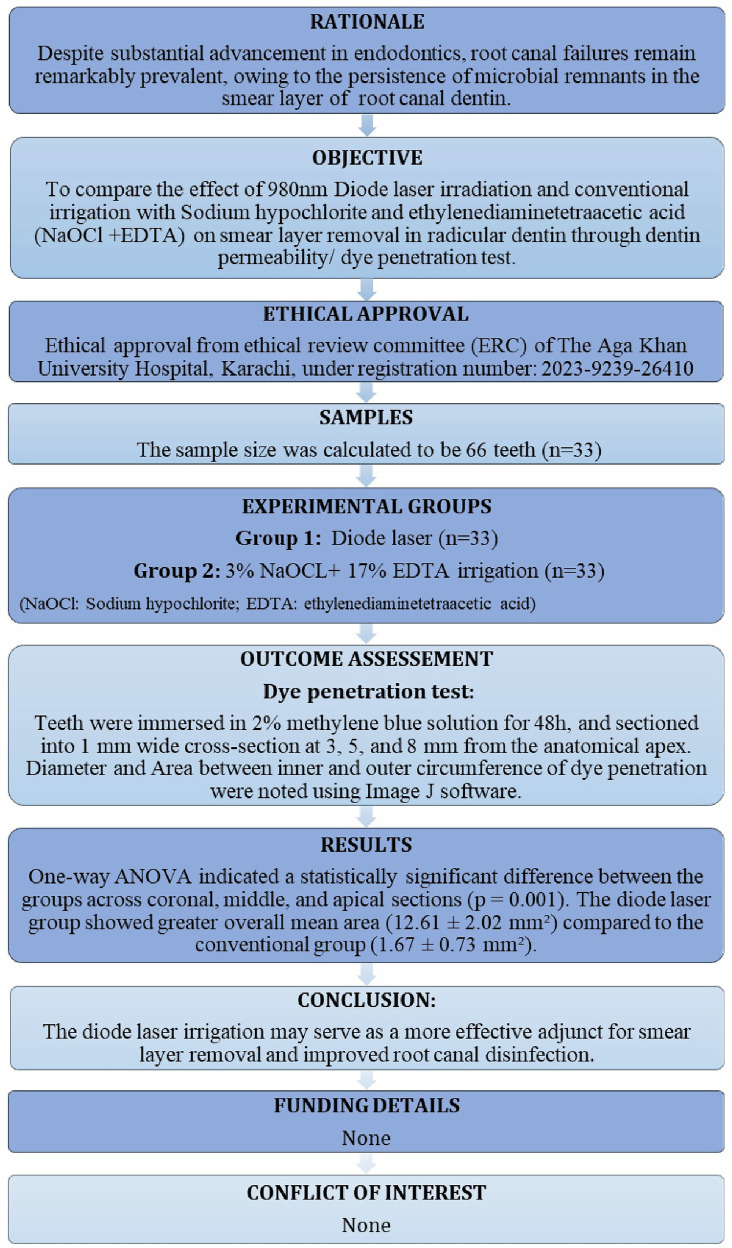
The PRILES flowchart. Preferred reporting items for laboratory studies (PRILES) in endodontology 2021 flowchart.

The present study was carried out in the Department of Operative Dentistry and Endodontics. The study design is in accordance with the Preferred Reporting Items for Laboratory studies (PRILES) in Endodontology 2021 and World Medical Association Declaration of Helsinki (2008) [Fig. 1] [20].

### Sample size

The sample size was determined using OpenEpi software version 3.0 (open-source statistics for public health, www.openepi.com) with the module for comparing two means. Based on the study by Al-Mafrachi et al [21]. the reported mean and standard deviation (SD) values of the area of dye penetration for smear layer removal using PDT and conventional irrigation were 48.3 ± 31.9 mm² and 71.6 ± 22.5 mm², respectively. With a 95% confidence interval and 90% power, the required sample size was calculated as 60 (*n* = 30). To account for potential sample loss during handling, the sample size was increased by 10%, yielding a final total of 66 samples equally distributed between the two groups *n* = 33(50%).

#### Inclusion criteria

The study included single-rooted extracted human teeth with sound structure and closed apices that were removed for reasons independent of this study. All teeth will have a Vertucci classification type 1 root canal configuration, i.e., a single canal confirmed by periapical digital radiographs with first file to bind at working length was atleast ISO K-files #25 [22, 23].

#### Exclusion criteria

Previously endodontically treated teeth and those with cracks, root fractures, calcified canals, root resorption, root caries, or immature apices were excluded.

### Data collection

Ultrasonic scalers (EMS 250 Piezon, Switzerland) were used to clean tooth surfaces of soft tissue, periodontal ligament remnants, and debris. The teeth were then immersed in 3% hydrogen peroxide for 5 min, followed by 0.2% thymol for 48 hours, and stored in 0.9% physiologic saline at 37 °C until further use, with storage not exceeding 4 weeks to prevent alterations in tooth properties. All sample preparation was performed by the primary investigator. When required, teeth were decoronated to standardize the root length to 15 mm. Straight-line access cavities were prepared using round diamond burs ISO 001/014 (Mani Dia BR-41, Mani, Japan) in a high-speed handpiece. After identifying the canal orifice and establishing a glide path, ISO K-files #10, #15, and #20 (Dentsply Maillefer, USA) were used up to working length, set 1 mm short of the anatomic apex. The first file to bind at working length were ISO K-files #25, #30, or #35, which determined the final finishing file size. Root canal shaping and finishing were performed using the ProTaper Next rotary system (X1 [17/0.04], X2 [25/0.06], X3 [30/0.07], and/or X4 [40/0.06]) at 300 rpm and 2.2 N/cm torque, as per the manufacturer’s instructions. Once instrumentation was completed, each tooth was assigned a unique number for identification and subsequently randomized into two groups using a computer-generated randomization list. The groups were then categorized based on the irrigation protocols to be evaluated in the study, ensuring equal and unbiased distribution of specimens between experimental and control conditions.

**Group I:** Diode laser irradiation

**Fig. 2:**
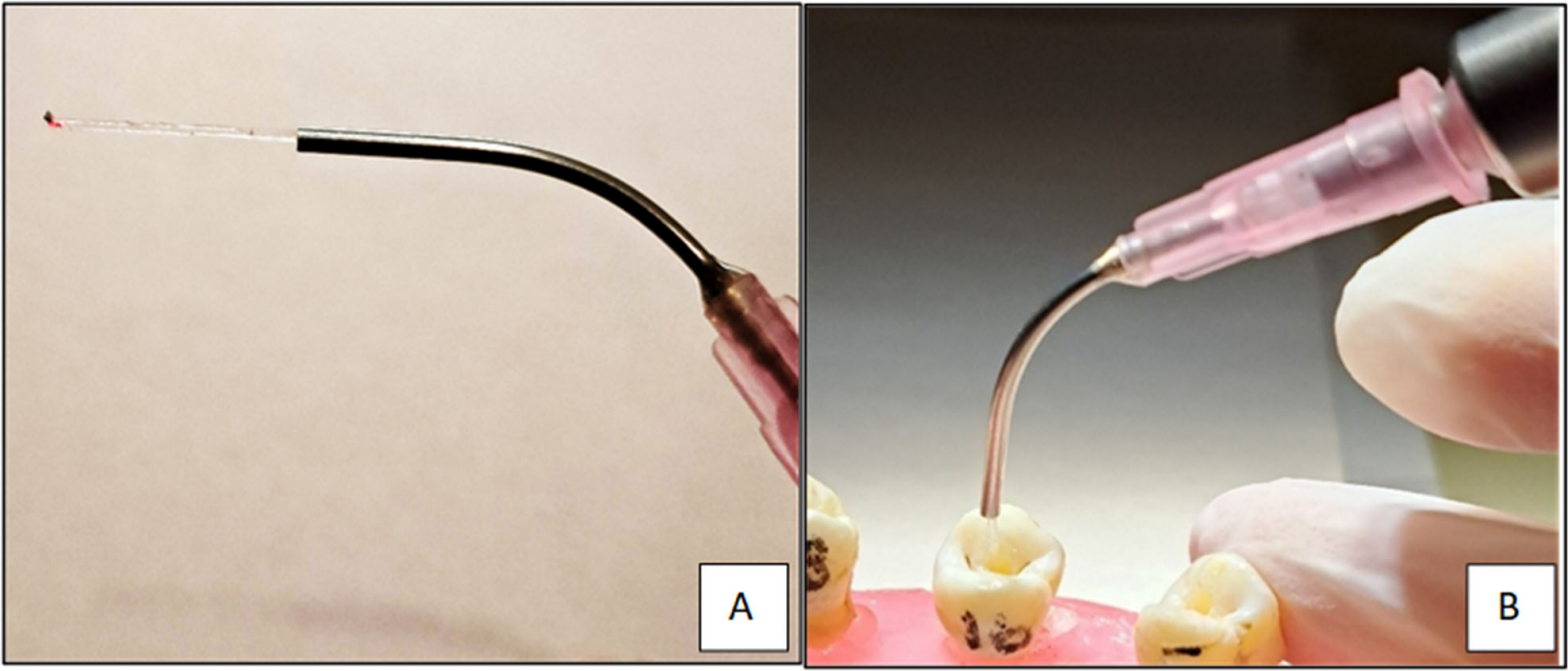
Application of Diode laser. **A** Diode laser 200 µm x 15 mm fiber-optic cable; **B** Cable inserted along the 1 mm from the radiographic apex of the root canal.

Irrigation was initially performed using 0.9% physiologic saline, after which laser-assisted activation was carried out with a 980 nm diode laser. The laser was operated at a wavelength of 980 nm, output power of 2 W, pulse duration of 1–4 ms, and a frequency of 200 Hz, using a 200 µm fiber-optic tip [21]. The fiber-optic tip was inserted up to 1 mm short of the radiographic apex (14 mm canal length), and irradiation was applied along all dentinal walls in a helical motion from apical to cervical at a speed of 1 mm/s. Each specimen was irradiated for 7 s, followed by a 20-s cooling period at room temperature to avoid excessive heat generation. The cycle was repeated four times, resulting in a total irradiation time of approximately 30 s per tooth, based on the mean root length of 15 mm [Fig. 2].

**Group II:** Conventional (3% NaOCl+ 17% EDTA irrigation)

The canals were irrigated with 3% NaOCl (Antiseptic Liquid #2, Technodent, Belograd, Russia) using 30-gauge × 25 mm side-vented needles (Irrigating Needle Tips, Henry Schein, NY, USA) after each successive filing. Final irrigation was performed with 10 mL of 17% EDTA (Coltene, USA) for 1 minute, followed by a rinse with 5 mL of NaOCl.

#### Dye penetration test

**Fig. 3:**
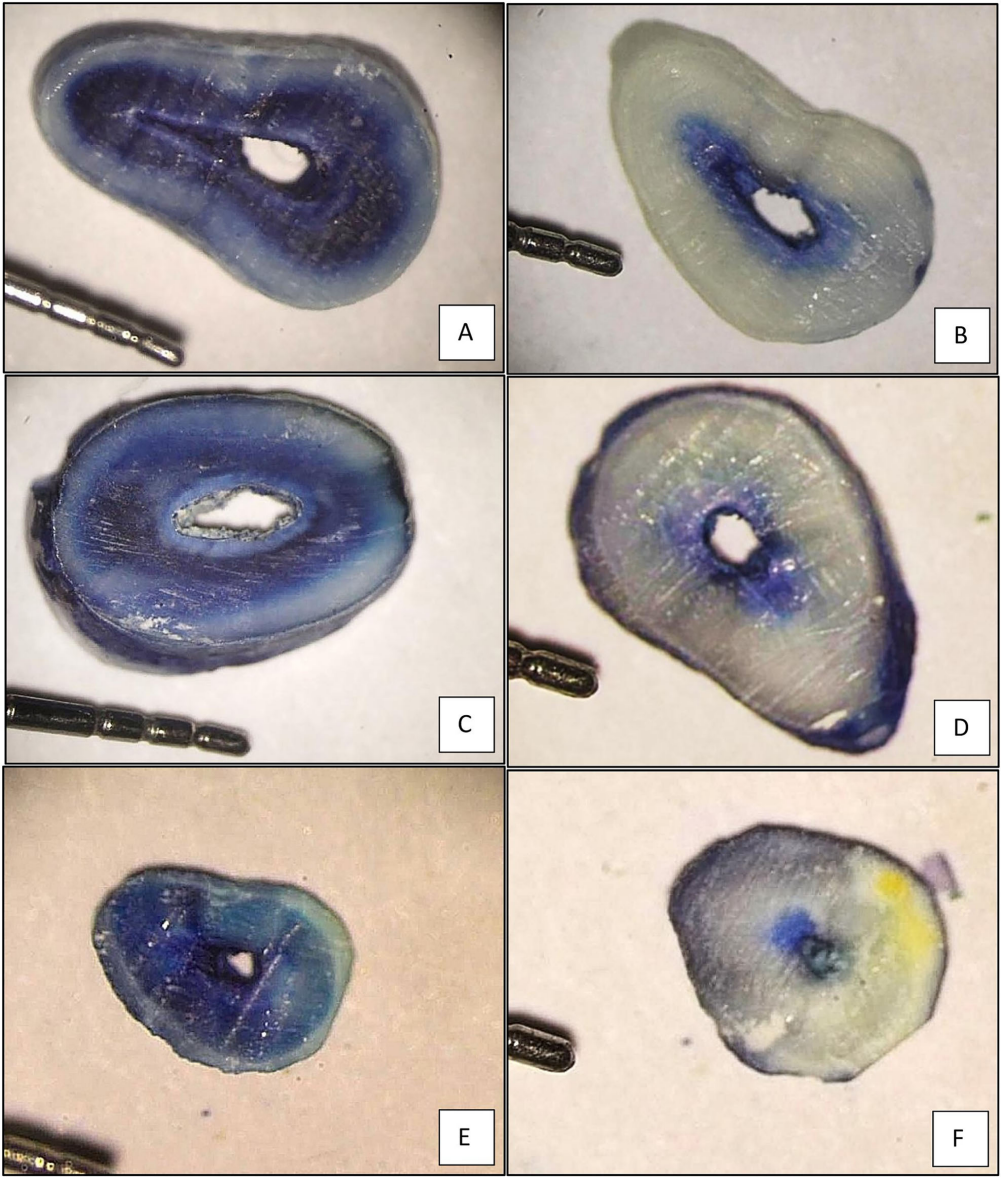
The Area of dye penetration. Comparison of dye penetration area in root cross-sections between the Diode laser group (**A**, **C**, **E**) and the NaOCl + EDTA group (**B**, **D**, **F**) at 8 mm, 5 mm, and 3 mm anatomical levels using stereo microscope (10–40x magnification).

All prepared teeth were coated with nail varnish to prevent external dye penetration, leaving only the canal orifice exposed. Following treatment, the canals were thoroughly dried using corresponding ProTaper Next paper points (Dentsply Sirona, USA) to ensure complete removal of moisture prior to dye application. The prepared root canals were then immersed in a 2% methylene blue solution for 48 hours to allow adequate penetration of the dye into dentinal tubules. After immersion, all samples were rinsed under running water to eliminate excess surface dye. Each tooth was subsequently embedded in self-cure acrylic resin blocks for stability during sectioning. Using a diamond bur mounted on a slow-speed handpiece with air-water coolant to prevent overheating, each specimen was sectioned into three horizontal slices. The sections were standardized to 1 mm thickness and obtained at 3 mm, 5 mm, and 8 mm from the anatomical apex, representing the apical, middle, and coronal thirds of the root canal, respectively [24]. The sections were examined by two independent evaluators (PI and Co-PI) under a stereomicroscope (AM-4000, ALLTION, Guangxi, China) at magnifications ranging between 10x and 40x. The digital images of each section were captured using a Canon EOS 4000D DSLR camera attached to the stereomicroscope. These images were then transferred to a computer and analyzed using ImageJ software (ImageJ software version 1.53 t), where the depth and area of dye penetration were measured with precision by the blinded examiner to the study objective and groups [Fig. 3] [21].

#### Smear layer scores

The diameter of the root canal lumen (inner measurement) and the circumference of dye penetration (outer measurement) were recorded in millimeters using ImageJ software. These values were used to assess the extent of dye infiltration within the dentinal tubules. The inner diameter represented the natural root canal lumen, while the outer circumference indicated the maximum extent of methylene blue penetration into the dentin from the canal walls. To quantify the overall penetration, the area of dye diffusion was calculated by determining the difference between the inner and outer diameters, which was then converted into squared millimeters (mm²).

### Statistical analysis

Data analysis was conducted using the Statistical Package for Social Sciences (SPSS; IBM version 23.0). Descriptive statistics, including mean and standard deviation, were calculated. To assess normality, both the Kolmogorov–Smirnov and Shapiro–Wilk tests were applied, confirming that the data followed a parametric distribution. For intergroup and intragroup comparisons of dye penetration across different root levels (coronal, middle, and apical thirds), one-way ANOVA was performed, followed by Bonferroni post-hoc correction to account for multiple comparisons and reduce the risk of Type I error. A significance level was maintained at ≤ 0.05 for all statistical tests. To ensure reliability in outcome assessment, two qualified examiners independently evaluated the specimens. Inter-examiner agreement was measured using Fleiss’ kappa statistic, which demonstrated excellent reliability with a value of 0.825 (95% CI: 0.820–0.829, *p* < 0.005). Both examiners were blinded to group allocation to minimize bias and strengthen the validity.

### Ethics

Declaration: The present study was carried out in the Department of Operative Dentistry and Endodontics at The Aga Khan University Hospital, Karachi, Pakistan. Ethical approval was obtained from the ethical review committee (2023-9239-26410).

## Results

### Diameter of dye penetration (mm)

Table 1 One-way ANOVA indicated a statistically significant difference in dye penetration diameter between the groups across coronal, middle, and apical sections (*p* = 0.001). Post-hoc Bonferroni analysis revealed that the diode laser group demonstrated overall significantly greater dentinal tubule penetration (16.2 ± 1.91) compared to the conventional group (5.32 ± 0.70; *p* = 0.001). Both groups showed the least penetration in the apical third, while the diode laser group achieved the highest penetration in the coronal third (23.96 ± 2.78) [Table 2].

**Table 1:** Diode laser parameters.

Specifications of diode laser	
Wavelength	980 nm
Power	2 watts
Pulse duration	1–4 ms
Frequency	200 Hz
Fiber optic size	200 µm

**Table 2:** Mean diameter (mm) of inner and outer circumference of dye penetration in cross section root relative to the apex.

Groups	Diameter of dye penetration in mm (mean ± SD)					*p*value
	Diameter	Coronal third 8 mm	Middle third 5 mm	Apical third 3 mm	Overall	
Group I (Diode laser)	Outer	23.96 ± 2.78	17.02 ± 2.07	7.62 ± 0.88	16.2 ± 1.91	0.001*ɸ
	Inner	7.24 ± 0.90	3.31 ± 0.53	0.21 ± 0.06	3.58 ± 0.49	
Group II (NaOCl + EDTA)	Outer	10.06 ± 1.12	5.34 ± 0.85	0.55 ± 0.14	5.32 ± 0.70	0.001*ɸ
	Inner	7.50 ± 0.48	3.21 ± 0.30	0.21 ± 0.05	3.64 ± 0.28	

Significance level *p* ≤ 0.05, *Statistically significant; ɸOne-way ANOVA (Bonferroni adjusted values).

*NaOCl* sodium hypochlorite, *EDTA* ethylenediaminetetraacetic acid, *SD*standard deviation.

### Area of dye penetration (mm^2^)

**Fig. 4:**
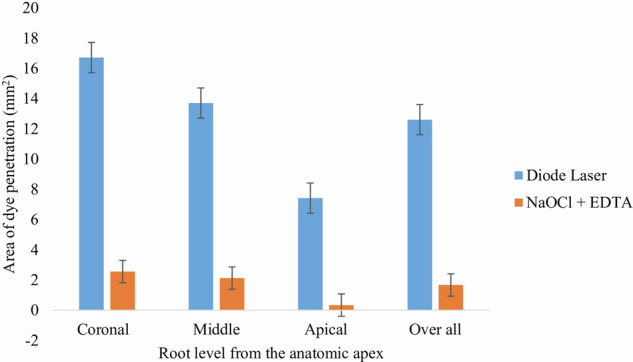
Mean area of dye penetration (mm²) at the coronal, middle and apical root levels, and overall, in the diode laser and NaOCl + EDTA groups. Error bars: standard deviation (NaOCl: Sodium hypochlorite; EDTA: ethylenediaminetetraacetic acid).

Statistical analysis demonstrated a highly significant difference between the root canal irrigants across all three levels (*p* = 0.001). The diode laser group showed a markedly greater overall mean area of dye penetration (12.61 ± 2.02) compared to the conventional group (1.67 ± 0.73). Intragroup analysis revealed significant differences across the root levels in both groups (*p* = 0.001 and *p* = 0.003), while intergroup comparisons confirmed highly significant differences at each corresponding level (*p* = 0.001) (Table 3, Fig. 4).

**Table 3:** Intergroup and intragroup comparison of area of dye penetration (mm^2^) across different root sections using independent sample *t* test.

Groups	Area of dye penetration in mm^2^ (mean ± SD)			Overall	*p*value (with in groups)
	Coronal third 8 mm	Middle third 5 mm	Apical third 3 mm		
Group I (Diode laser)	16.72 ± 3.04	13.71 ± 2.15	7.412 ± 0.87	12.61 ± 2.02	0.001*ɸ
Group II (NaOCl + EDTA)	2.56 ± 1.30	2.13 ± 0.74	0.342 ± 0.161	1.67 ± 0.73	0.003*ɸ
*p*value (between groups)	0.001*	0.001*	0.001*	0.001*	

Significance level *p* ≤ 0.05, *Statistically significant; ɸOne-way ANOVA (Bonferroni adjusted values).

*NaOCl* sodium hypochlorite, *EDTA* ethylenediaminetetraacetic acid, *SD* standard deviation.

## Discussion

The present study demonstrated greater area of dye penetration in the diode laser group (12.61 ± 2.02) compared to the conventional NaOCl + EDTA group (1.67 ± 0.73) across the different sections of the root canal. These findings suggest that diode laser irradiation enhances smear layer and plug removal and increases dentinal patency and permeability, allowing deeper penetration of irrigants and potential medicaments. Thereby, the null hypothesis was rejected.

The superior performance of diode lasers can be explained by their combined thermal and photomechanical effects, which effectively disrupt smear layer components. These laser emit light in the near-infrared spectrum, where their energy is absorbed by the fluids within the canal; the resulting heat promotes the generation of reactive oxygen species (ROS) and partially vaporize and modifies the smear layer, thereby enhancing dentinal permeability [14]. The dye penetration area of the present study were consistent to the study conducted by Al Mafrachi et al. [21] with 940 nm wavelength of diode laser. Also, he reported minimal to no residual smear layer under scanning electron microscopy, with clearly visible and open dentinal tubules compared to conventional irrigation [21]. In contrast, Dhawan et al. [25] found no significant difference between 810 nm diode laser and chemical irrigation [25]. One plausible explanation is that the use of different laser parameters may account for the conflicting results in various studies.

The laser used in the present study has a wavelength of 980 nm that offers an irradiation distance of up to 0.5–3.5 mm, enabling greater intratubular penetration even in deeper root canal regions that are otherwise difficult for conventional irrigants to access [26]. Notably, diode lasers have a relatively low absorption coefficient in hard dental tissues such as enamel and dentin [27]. This reduces the risk of excessive surface ablation compared to other laser types used in previous studies [28]. However, if the laser parameters are not carefully controlled, the deeper energy transmission can lead to excessive heat generation, and prolonged or high-power application may result in structural alterations of dentin, such as melting, cracking, or surface carbonization [27, 28].

Conventional irrigation protocol using NaOCl and EDTA demonstrated lesser dentin tubule patency as compared to diode laser in the present study. The lower dye penetration values observed are consistent with the previous literature [3, 25, 29]. The performance of NaOCl and EDTA is highly dependent on key variables such as concentration, pH, and duration of exposure [30, 31]. Prolonged contact or inappropriate concentrations can negatively influence the structural integrity of dentin; disproportionate dissolution of the organic and inorganic phases of dentin can weaken its mechanical properties [30, 31]. These drawbacks highlight the importance of optimizing irrigation protocols to balance effective smear layer removal with preservation of structural integrity. A study conducted by Sabah M [32]. demonstrated that activation of 2.6% NaOCl and 17% EDTA with a diode laser significantly improved the quality of the root canal seal and the adaptability of filling materials to the canal walls when examined under scanning electron microscopy (SEM). This suggests that combining laser activation with chemical irrigation yields superior outcomes compared to using the laser alone, as the synergistic effect enhances smear layer removal, promotes deeper irrigant penetration and improves dentinal permeability. Moreover, recent studies have shown that laser activation of chemical irrigants enhances their antibacterial effectiveness and improves smear layer removal compared with irrigation or irradiation alone [33–36].

In the present study, the greater dye penetration was observed in the coronal and middle thirds compared to the apical third in the conventional group; this can be attributed to anatomical variations and difficulties in achieving effective irrigant delivery at apical levels. The previous studies observed that apical dentin generally exhibited inferior outcomes compared to the coronal and middle thirds [32]. This may be explained by findings from SEM analysis in a previous study, which reported that dentinal tubule diameters were larger in the coronal (4.32 μm) and middle (3.74 μm) zones compared to the apical zone (1.73 μm) [37–39]. Also, the lower number of dentinal tubules, the high mineral content and the presence of sclerotic dentin in the apical part can reduce the effectiveness of the irrigation [32, 37]. Nonetheless, the diode laser was able to significantly improve penetration even in the apical third, highlighting its potential advantage in areas that are typically challenging to disinfect. These findings are consistent with the studies by Parirokh M et al. [31] and Amin K et al. [32] which reported that optimal disinfection could be achieved in the apical region of the canal through diode laser irradiation.

The interaction between the initial laser-induced acoustic waves and those reflected from the canal walls can vary depending on the size and taper of the prepared root canal [40]. In this study, the canals were prepared using the ProTaper Next rotary file system. For optimal laser-activated irrigation, the recommended root canal taper is approximately 0.04–0.06, as this dimension facilitates sufficient space for safe fiber tip insertion without binding against canal walls [41]. Typically, a 200–300 µm fiber tip is employed with diode lasers, and preparation to at least an apical size of #25–35 is advised to ensure homogeneity and effective delivery of laser energy closer to the apical third, thereby maximizing disinfection and smear layer removal while minimizing the risk of procedural errors [40, 41]. In the present study no photoactive dyes were used. Previous literature discourages the use of photoactivated dyes due to their chromogenic potential, as they can penetrate enamel and dentin during irrigation or photodynamic therapy and result in visible tooth staining [42]. While some discoloration may be temporary or removable, deeper penetration into dentinal tubules can lead to residual tooth discoloration [42].

Methylene blue used in the present study serves as an effective tracer dye to assess the depth and extent of irrigant or medicament penetration into dentinal tubules. Its small molecular size (1.43 nm) and strong affinity for dentin allow it to penetrate tubules effectively [43]. Compared to fluorescent dyes or radiolabeled markers, methylene blue is inexpensive, safe, and widely accessible. It is non-toxic at the concentrations typically used for laboratory studies, making it suitable for in vitro research.

### Limitations

Within the limitations of the study, increased dye penetration indicates improved permeability; however, further research is necessary to determine whether this improvement translates into superior microbial elimination, long-term preservation of dentin integrity, and improved clinical outcomes as compared to other lasers and ultrasonic irrigation protocols. As this was an in vitro investigation, the experimental conditions may not fully replicate clinical scenarios, and the findings should therefore be interpreted with caution. Future research should emphasize advanced assessment tools and techniques, microbial-based evaluations and well-designed clinical trials to validate these results and establish their clinical relevance.

## Conclusion

The diode laser achieved greater patency of dentinal tubules compared to the conventional NaOCl + EDTA group across all root levels. These findings suggest that diode laser irradiation may serve as a effective adjunct for smear layer removal and improved root canal disinfection. Future research should explore the effect of different types of lasers and chemical irrigants to better understand their interaction with laser-assisted irrigation techniques.

## Data Availability

All data generated or analyzed during this study are included in this published article. Additional details, if required, are available from the corresponding author upon reasonable request.
